# Adolescent Girls’ Weight-Related Family Environments, Minnesota

**Published:** 2011-04-15

**Authors:** Katherine W. Bauer, Dianne Neumark-Sztainer, Mary Story, Jayne A. Fulkerson

**Affiliations:** Division of Epidemiology and Community Health, University of Minnesota; University of Minnesota, Minneapolis, Minnesota; University of Minnesota, Minneapolis, Minnesota; School of Nursing, University of Minnesota, Minneapolis, Minnesota

## Abstract

Significant sociodemographic disparities exist in the prevalence of obesity among adolescent girls, and in girls' participation in physical activity, sedentary activity, and healthful dietary intake. However, little is known of how factors in the family environment associated with weight and behavior vary by sociodemographic groups. We examined differences and similarities in the weight-related family environments of adolescent girls by race/ethnicity, parental educational attainment, and US nativity. Data are from the baseline assessment of 253 parent/daughter dyads. Parents completed survey items on the family environment; parents and girls reported their sociodemographic characteristics. Hierarchical regression models were used to test relationships between the family environment and sociodemographic characteristics. Parents of Asian girls reported qualities supportive of physical activity and healthy eating. Higher parental education was associated with more parental modeling of and support for physical activity and greater frequency of family meals. Parents of foreign-born girls reported having fewer televisions in the home, more frequent family meals, and fewer fast-food family meals. Understanding sociodemographic differences in the family environments of adolescent girls can inform the development of obesity prevention programs and reduce disparities in adolescents' weight status, physical activity, sedentary behavior, and healthful dietary intake.

## Introduction

More than one-third of adolescent girls are overweight or obese, and large sociodemographic disparities have been observed both in the prevalence of overweight and obesity among girls and in girls' participation in physical activity, sedentary behavior, and healthful diet (1-3). A growing body of evidence has demonstrated the role of the family environment in adolescents' weight and weight-related behavior (4-6). Factors included are parental modeling and encouragement of physical activity, television use, and healthful diet, and resources available in the home such as fruits and vegetables. However, little is known about how families from varied sociodemographic backgrounds may differentially support or inhibit girls' healthful behavior. Identifying differences in adolescent girls' weight-related family environments by sociodemographic characteristics can help guide the targeting and tailoring of obesity prevention efforts to specific populations.

A small number of studies have identified differences in the family environment relevant to adolescents' physical activity, television use, and dietary behavior by sociodemographic characteristics (7-10). Although these studies identified differences between families by race/ethnicity and socioeconomic status (SES), all examined a limited number of family environment characteristics and all but 1 used adolescents' report of the family environment rather than the parents' report. Parents' report of their own behavior and the resources available in their home may be more accurate than adolescents' report (8,11) and can be informative for the development of interventions targeting parents. Therefore, our goal was to examine how weight-related factors in adolescent girls' family environments differ by girls' race/ethnicity, US nativity, and parental education, a commonly used indicator of SES (9,12).

## Methods

### Study design

This cross-sectional study used data from the baseline assessment of adolescent girls in Minnesota who participated in the New Moves intervention during either the 2007-2008 or 2008-2009 school year. New Moves is a school-based physical activity and nutrition intervention implemented primarily through an all-girls physical education class (13). Girls were in grades 9 through 12 and had a mean age of 15.7 years (range, 12.0-20.3). Schools that participated in the study were in urban and first-ring suburban areas.

On average, 56% of students in the 12 schools that participated in the study were eligible for free or reduced-price school breakfast and lunch. The New Moves intervention was advertised to all girls in the school, and recruitment materials were designed to appeal to girls who were inactive and not comfortable being physically active, but who had a desire to be healthier. Girls were excluded from the study if they reported participating in more than 1 hour of physical activity per day or reported eating disorder behavior (vomiting or laxative use more than 1 time per week) on the basis of a short screening questionnaire. Parents of the 356 girls who participated in the New Moves study were asked to complete a survey assessing the family environment at baseline; 71% responded, resulting in 253 parent-girl dyads. Three of these parent-girl dyads were excluded because of missing sociodemographic data. Girls completed study measures at either a university clinic or their high school. Parents were mailed a recruitment packet including the family environment survey after their daughter enrolled in New Moves. Most parents completed the survey and returned it by mail, although 2% of parents completed the survey by telephone with the assistance of research staff. University of Minnesota's institutional review board and each participating school district approved the study. Girls and parents provided consent or assent for their participation in the study.

### Measures

Survey items completed by parents assessed parental modeling of and support for girls' physical activity, television use, soft drink consumption, fruit and vegetable consumption, and home resources associated with these behaviors (Appendix). Most of the items have been used in previous studies, and many have been tested for validity and reliability, or both, and psychometric information is reported when available.

Parental education was determined by the question, "How far did you go in school? (Indicate highest level)." Response options were "did not finish high school," "finished high school or got GED (general educational development certificate)," "did some college or training after high school," "graduated from a college or university," and "professional training beyond a 4-year college degree." The highest 2 categories were combined in analyses to create a "college and postgraduate training" category.

Girls' race/ethnicity was assessed by girls' response to the item, "Do you think of yourself as . . .". Response options were white, black or African American, Asian, Native Hawaiian or other Pacific Islander, American Indian or Alaska Native, Hispanic or Latina, and mixed/other. Girls could select all categories that applied to them. If a girl selected 2 categories including white, she was categorized as the nonwhite race/ethnicity. If a girl selected 2 racial/ethnic categories that did not include white or selected 3 or more categories, she was included in the mixed/other category. Due to the small number, girls who selected American Indian or Alaska Native were included in the mixed/other category. Girls' US nativity was assessed with their response to the question, "Were you born in the United States?" (yes/no).

### Statistical analysis

Separate hierarchical multiple regression models were used to examine the relationships between families' sociodemographic characteristics and family environment constructs. Two regression models were developed: 1 with only the sociodemographic characteristic of interest as the predictor, and 1 with all sociodemographic characteristics simultaneously entered into each model to obtain the independent association between the characteristic of interest and the family environment component. The patterns of relationships between the sociodemographic characteristics and the family environment variables were similar in both the single predictor and mutually adjusted analyses; therefore, only the results from the adjusted analyses are presented.

To account for potential clustering of family environment factors among girls who attended school together, a school-level variable was included in the regression models as a random effect. Adjusted means were calculated for each level of each sociodemographic characteristic. If the *F* statistic was significant, an adjusted Tukey-Kramer test was used to highlight sources of differences between the adjusted means. A linear test of trend was used to determine whether there was a significant linear relationship between the family environment components over the 4 levels of parental education. In our study, at 80% power, a difference of 0.5 standard deviations could be detected between typical subgroups of approximately 65 people. We used SAS version 9.2 (SAS Institute, Inc, Cary, North Carolina) to conduct analyses.

## Results

The study sample was racially and ethnically diverse: 30% of girls reported that they were white; 26%, African American/black; 11%, Hispanic; 24%, Asian; and 10% of mixed race or another racial/ethnic group. Among Asian girls, 87% self-identified as Hmong. Parents' education was also diverse: 28% of parents had not completed high school, 21% had only a high school diploma, 26% attended some college, and 25% completed college or had postgraduate training. Approximately one-fourth (23%) of the girls were foreign-born. Mothers were the most common respondents to the parent survey (80%).

### Family environment by girls' race/ethnicity

Several elements of the family environment differed by girls' race/ethnicity after adjustment for parental education and US nativity (Table 1). Overall, parents of Asian girls were most likely to provide a family environment supportive of physical activity and healthful eating. Asian girls were the least likely to have a television in their bedroom, and their parents reported watching television the least frequently, having the least amount of unhealthy food in their home, consuming the most servings of fruits and vegetables, and consumed the least number of soft drinks compared with parents of girls of other race/ethnicities. Overall, parents of Hispanic girls and girls of mixed/other race, and to a lesser extent, parents of African American and white girls, reported fewer family environment characteristics that are supportive of physical activity, healthful diet, and limiting television use. For example, parents of African American and Hispanic adolescents reported the fewest number of physical activity resources in the home, and Hispanic parents and parents of girls of mixed/other race consumed soft drinks the most frequently.

### Family environment by parental education

Girls' family environments also differed by parental education after adjustment for race/ethnicity and US nativity (Table 2). All of the physical activity–related family environment components were positively associated with parental education, including availability of physical activity resources, parental modeling of moderate-to-vigorous physical activity (MVPA), and family support for their daughter's physical activity. Several elements of the family environment related to food behaviors were associated with parental education, including less parental modeling of soft drink intake and greater frequency of family meals. Parents who did not complete high school reported having family meals 3.8 times per week, compared with 5.4 times per week among parents with a college degree or higher. The percentage of girls with televisions in their bedrooms was associated with parental education. Girls with parents who had either the lowest or highest level of education were less likely to have a television in their bedroom than girls with parents who either completed high school or had some college.

### Family environment by girls' nativity

Parents of foreign-born girls reported several differences in their family environment compared with parents of girls who were born in the United States, after controlling for race/ethnicity and parental education (Table 3). For example, parents of foreign-born girls reported having fewer televisions in their home and having more frequent family meals. In contrast, parents of foreign-born girls were less likely to report providing support for their daughters' physical activity and healthful eating.

## Discussion

Although many aspects of girls' weight-related family environments were similar across sociodemographic groups, several noteworthy differences emerged. Overall, findings suggest that parents of Asian girls, parents with higher education, and parents who have immigrated to the United States since their daughter's birth may be more able to provide their daughters with a family environment supportive of physical activity and healthful diet, and limiting of sedentary behavior. Understanding these sociodemographic differences in the weight-related family environments of youth aids in the development of interventions to address families' specific needs and, ultimately, in the reduction of sociodemographic disparities in obesity among adolescents.

Specifically, parents of Asian girls reported providing a more health-promoting environment for their daughters. They were less likely to have a television in their daughters' bedroom, had less unhealthful food in the home, and modeled greater consumption of fruits and vegetables and less consumption of soft drinks. Using nationally representative data, Gordon-Larsen et al (21) found that Asian girls reported viewing significantly fewer hours of television than African American and Hispanic adolescent girls. This racial disparity in girls' television use may in part be due to the differences identified in our study, including less parental modeling of television use and fewer televisions available in the homes of Asian girls. These findings suggest that obesity prevention interventions that aim to modify the family environment and help both parents and adolescents improve their physical activity and diet may see the greatest benefit from recognizing diversity in families' cultural norms around supporting physical activity, television use, and healthful diet and developing intervention components that are culturally appropriate and relevant.

Parental education was positively associated with the presence of health-supportive factors in the family environment, including parental modeling of and support for physical activity and healthful eating and having frequent family meals. The associations found between parental education and home physical activity resources, parental physical activity, and parental soft drink consumption are similar to the findings of previous studies that noted that families with higher parental education or SES provided multiple resources to support their children's physical activity and healthful eating (9). The positive associations found between parental education and a supportive family environment may aid in understanding the findings of other studies that adolescents whose parents have higher levels of education engage in more physical activity and have healthier dietary habits (22).

Despite evidence that adolescents whose parents have lower education are more likely to watch television than adolescents with parents of higher education (23), we saw few associations between parental education and factors in the family environment related to television use. Therefore, differences in adolescents' television use by SES may be attributable to factors in the home or outside of the home not assessed in the current study, such as amount of unsupervised time at home or lack of community-based opportunities for adolescents after school or on weekends in low-SES neighborhoods. In light of the associations observed between adolescents' television use and weight (24), research is needed to understand the factors that contribute to excessive television use among lower SES youth in order to develop policies and programs that encourage adolescents to be physically active in their leisure time.

One notable observation from our study is the nonlinear relationship between parental education and the presence of a television in girls' bedrooms. This finding differs from that of a previous study in which adolescents whose parents had less education were more likely to have a television in their bedroom compared with adolescents whose parents had greater education (7). The low prevalence of a television in the bedroom at both the low and high levels of parental education seen in our study may reflect different forces: families from lower-SES backgrounds may find televisions too expensive, and parents of higher education may be more likely to heed warnings to restrict televisions in children's bedrooms. Despite increasing efforts to inform parents of the detrimental effect that excessive television use can have on children's health, parental encouragement for their daughters to limit television use was low and did not differ by any of the sociodemographic characteristics we assessed. Previous studies have observed that the presence of familial rules and restrictions on television use are associated with less television use among younger children (25), but parents of increasingly independent adolescents may need alternative strategies to limit their children's television use.

Findings that parents of girls born outside the United States provide many resources to support their daughters' participation in physical activity and healthful dietary practices align with previous research that showed that adolescents of white, African American, and Asian descent born outside the United States were less likely to watch excessive television (26), and many adolescents born outside the United States consume more healthful diets (27) than their US-born peers. In contrast, families of foreign-born girls were less likely to report providing explicit support for their daughter's physical activity and healthful eating. Further research is needed to understand the cultural, social, or economic mechanisms that contribute to these findings. Understanding why certain families choose not to or are unable to provide logistic or emotional support for their children's health behaviors may guide interventions to help such families. However, as a whole, these results indicate that practitioners need to support the families of recent immigrants in maintaining a resource-rich family environment to avoid the development of unhealthy weight-related behaviors that foreign-born adolescents often adopt as they acculturate to US society (28).

This is one of the few studies that has collected data on the weight-related family environment from parents rather than from adolescents (29). Parents' and adolescents' reports of their family environment have been found to be discrepant (30), and it has been hypothesized that parents may provide a more valid assessment of their own behavior and other aspects of the family environment such as food purchases (8). Additionally, parents' perceptions of the family environment play a critical, yet less understood role in adolescents' behavior, and data collected from parents can be used to develop programs to help parents improve their family environment.

A limitation of our study is that parent education was the only marker of family SES, which may not fully reflect a families' socioeconomic position. The inclusion of multiple measures of SES, including parental occupation and household income, would have allowed for a more complete understanding of girls' family environments. Additionally, differences observed in parents' report of the family environment by sociodemographic characteristics may reflect differences in understanding or interpretation of survey items. This may be especially true for parents who are not proficient in English. Future research exploring family environments may benefit from qualitative research and extensive pilot testing to ensure that survey items are appropriate and valid for the intended population; offering participants the ability to complete study measures in their native language could also aid research. Finally, social desirability bias may have been differential across sociodemographic groups, potentially biasing study findings.

Overall, our study identified several differences in adolescent girls' family environments by race/ethnicity, parental education, and US nativity. These findings suggest that interventions that aim to help parents support and model healthful behavior for their adolescents, as well as increase resources in the home that aid in healthful eating and dietary behaviors, would benefit from targeting specific populations that have the most potential for improvement and from tailoring intervention materials to the needs of those families. Additionally, identifying population subgroups that provide a family environment that is supportive of physical activity and healthful eating behaviors and discourages sedentary behavior, such as Asian families and families who recently immigrated to the United States, can help elucidate what resources other families may need to maintain or develop a health-promoting family environment.

## Figures and Tables

**Table 1:** Differences in the Weight-Related Family Environments of Adolescent Girls (n = 250), by Race/Ethnicity, Minnesota

Weight-Related Characteristic	Adjusted Means^a^					*F*_4_	*P* Value

	White (n = 74)	African American/Black (n = 65)	Hispanic (n = 27)	Asian (n = 60)	Mixed/Other (n = 24)		
**Family physical activity environment**							
Physical activity resources (range, 0-9)^b^	4.4^x^	2.8^y^	2.8^yz^	3.6^z^	3.5^yz^	5.8	<.001
Parental moderate-to-vigorous physical activity, h/wk	3.6	2.9	3.0	3.4	2.9	0.5	.77
Family support for physical activity (range, 5-15)	12.2	12.5	12.9	13.1	14.0	0.7	.59
**Family television use**							
Media resources (range, 0-5)	4.2	3.6	3.9	3.8	3.7	1.7	.16
Televisions in the home (range, 0-≥4)	2.8	3.0	3.0	2.6	3.1	1.7	.16
Girls with television in bedroom, %^b^	46.3^x^	56.5^x^	48.3^x^	14.1^y^	79.3^z^	10.2	<.001
Parental television use, h/wk^b^	15.2^xy^	18.6^xy^	22.5^x^	14.7^y^	17.7^xy^	3.0	.02
Familial encouragement to limit television use (range, 1-5)	2.7	2.9	3.4	3.4	3.1	2.3	.06
**Family food environment**							
Healthful home food availability (range, 4-16)	12.0	11.4	12.3	11.9	11.3	0.9	.48
Unhealthful home food availability (range, 4-16)^b^	9.6^xy^	8.91^xy^	10.0^x^	8.3^y^	9.8^xy^	3.0	.02
Parental fruit and vegetable intake, servings/d^b^	4.2^x^	5.3^xy^	4.7^xy^	6.1^y^	5.0^xy^	3.0	.02
Parental soft drink intake, servings/wk^b^	1.5^xy^	2.0^yz^	2.3^z^	1.0^x^	2.7^z^	5.3	<.001
Familial encouragement for healthful eating (range, 1-5)	3.8	3.9	4.1	3.8	4.1	0.4	.80
Family meals, times/wk	4.8	4.1	4.9	4.7	4.7	0.6	.70
Fast-food family meals, times/wk	1.2	1.6	1.7	1.5	1.4	0.6	.67

a Parental education and girls' foreign-born status included as covariates, school included as random effect.

b Within this row, adjusted means that share a common superscripted letter (x, y, or z) are not statistically different at an α level of .05. Adjusted means that do not share a common superscripted letter are statistically different.

**Table 2:** Differences in the Weight-Related Family Environments of Adolescent Girls (n = 250), by Parental Education, Minnesota

Weight-Related Characteristic	**Adjusted Means** a				Linear Estimate	*P* Value for Trend

	<High School (n = 69)	Completed High School (n = 52)	Some College (n = 66)	College and Post-graduate Training (n = 63)		
**Family physical activity**						
Physical activity resources (range, 0-9)	2.9	3.4	3.8	4.2	4.4	<.001
Parental moderate-to-vigorous physical activity, h/wk	2.5	2.4	4.2	3.7	5.4	.008
Family support for physical activity (range, 5-15)	11.6	12.4	13.2	13.8	7.5	.01
**Family television use**						
Media resources (range, 0-5)	3.5	4.0	4.0	4.0	1.3	.09
Televisions in the home (range, 0-≥4)	2.6	3.1	2.9	2.9	0.8	.22
Girls with television in bedroom, %^b^	39.9^xy^	54.7^x^	56.7^x^	28.7^y^	10.2^c^	.003^c^
Parental television use, h/wk	16.0	19.4	17.3	15.6	−3.1	.64
Familial encouragement to limit television use (range, 1-5)	2.9	3.1	3.0	3.0	0.3	.77
**Family food environment**						
Healthful home food availability (range, 4-16)	11.7	11.4	11.7	12.4	2.7	.11
Unhealthful home food availability (range, 4-16)	9.5	9.3	9.0	8.8	−2.1	.20
Parental fruit and vegetable intake, servings/d	4.4	5.2	5.5	5.2	2.7	.12
Parental soft drink intake, servings/wk	1.8	2.6	1.6	1.1	−3.2	.005
Familial encouragement for healthful eating (range, 1-5)	3.6	4.0	4.0	4.1	1.5	.04
Family meals, times/wk	3.8	4.3	4.9	5.4	5.4	.006
Fast-food family meals, times/wk	1.3	1.7	1.5	1.3	0.1	.93

a Girls' race/ethnicity and foreign-born status included as covariates, school included as random effect.

b Within this row, adjusted means that share a common superscripted letter (x, y, or z) are not statistically different at an α  level of .05. Adjusted means that do not share a common superscripted letter are statistically different.

c Due to the nonlinear association observed between parental education and the prevalence of a television in girls' bedrooms, the estimate presented is an *F* statistic from an analysis of variance model.

**Table 3:** Differences in the Weight-Related Family Environment of Adolescent Girls (n = 250) US Nativity,  Minnesota

Weight-Related Characteristic	**Adjusted Means** a		*F*_1_	*P* Value

	US Born (n = 192)	Foreign Born (n = 58)		
**Family physical activity**				
Physical activity resources (range, 0-9)	3.8	2.9	5.6	.02
Parental moderate-to-vigorous physical activity, h/wk	3.2	3.3	0.1	.83
Family support for physical activity (range, 5-15)	13.3	10.9	8.4	.004
**Family television use**				
Media resources (range, 0-5)	3.9	3.7	0.6	.44
Televisions in the home (range, 0-≥4)	3.0	2.3	19.6	<.001
Girls with television in bedroom, %	46.0	40.2	0.5	.47
Parental television use, h/wk	17.9	14.0	4.3	.04
Familial encouragement to limit television use (range, 1-5)	3.1	2.8	1.4	.24
**Family food environment**				
Healthful home food availability (range, 4-16)	12.0	9.1	2.4	.12
Unhealthful home food availability (range, 4-16)	9.2	11.3	0.1	.76
Parental fruit and vegetable intake, servings/d	5.1	5.0	0.01	.92
Parental soft drink intake, servings/wk	1.9	1.3	3.0	.08
Familial encouragement for healthful eating (range, 1-5)	4.0	3.6	5.0	.03
Family meals, times/wk	4.3	5.7	7.1	.008
Fast-food family meals, times/wk	1.6	1.0	4.1	.04

a Girls' race/ethnicity and parental education included as covariates, school included as random effect.

## Appendix. Parent Survey of Family Environment Constructs and Measures

**Table:**

**Family Environment** Constructs	**Survey Item**	**Psychometric information**
**Family physical activity**		
Physical activity resources	Please indicate which items you have in your home, yard, or apartment complex that are available to your daughter: stationary aerobic equipment (bicycle, treadmill, etc.) bicycle dog to walk weight lifting equipment (free weights, Nautilus, etc.) exercise workout videotapes or DVDs in-line, roller, or ice skates sports equipment (balls, racquets, jump ropes, hula hoops) skis or snowboard stretching or yoga equipment Response options included Yes and No (14)
Parental modeling of moderate-to-vigorous physical activity	In the past week (7 days), how many HOURS did you spend doing the following activities? Strenuous exercise (heart beats rapidly) Examples: biking fast, aerobic dancing, running, jogging, swimming laps, rollerblading, skating, tennis, cross-country skiing, soccer, basketball Moderate exercise (not exhausting) Examples: walking quickly, dancing, baseball/softball, gymnastics, easy bicycling, volleyball, strength training Nine response options ranged from 1 to 7 or more hours (15)	2-week test-retest *r* = .48-.94
Family support for physical activity	During a typical week, how often have you or another member of your household: encouraged your daughter to do physical activities or play sports? done a physical activity or played sports with your daughter? provided transportation to a place where your daughter can do physical activities or play sports? watched your daughter participate in physical activities or sports? told your daughter that she was doing well in physical activities or sports? Five response options ranged from "Never" to "Every day" (4)	Cronbach α = .78 1-week test-retest *r* = .81
**Family television use**		
Media resources	Please indicate which of the following you have in your home: Pay television (cable, satellite, etc.) Video/DVD player Electronic game (Nintendo, Playstation, etc.) Computere Internet access Response options included Yes and No (16)
Televisions in the home	How many televisions do you have in your home? Response options ranged from "0" to "4 or more" (16)	test-retest *r* = .95
Television in girls' bedroom^a^	Do you have a television in the room where you sleep? Response options included Yes and No (7)	
Parental television use	On a typical weekday (Monday through Friday), how many hours do you spend doing the following? Watching TV/Videos/DVDs On a typical weekend day (Saturday and Sunday), how many hours do you spend doing the following? Watching TV/Videos/DVDs Seven response options ranged from "0 hr" to ">5 hr" (17)	
Familial encouragement to limit television use	During a typical week, how often have you or another member of your household encouraged your daughter to watch less TV? Five response options ranged from "Never" to "Every day"	
**Family food environment**		
Healthful home food availability	In the past 7 days vegetables were available in my home vegetables were served at meals in my home fruit was available in my home fruit was served at meals in my home Four response options ranged from "Never" to "Always" (11)	Cronbach α = .63 2-week test-retest *r *= .54-.59
Unhealthful home food availability	In the past 7 days regular soda pop or other sugar-sweetened drinks were available in my home regular soda pop or other sugar-sweetened drinks were served at meals in my home chips or other salty snacks were available in my home candy was available in my home Four response options ranged from "Never" to "Always" (11)	Cronbach α = .80 2-week test-retest *r* = .55-.72
Parental fruit and vegetable intake	Thinking back over the PAST WEEK, how many servings of FRUIT did you USUALLY eat on a typical day? A serving would be a medium piece of fruit or ½ cup of fruit. Do not include fruit juice. Thinking back over the PAST WEEK, how many servings of VEGETABLES did you USUALLY eat on a typical day? A serving would be a ½ cup of cooked vegetables or 1 cup of raw vegetables. Do not include potatoes or french fries. Seven response options ranged from "None" to "5 or more servings" (18)	
Parental soft drink intake	Thinking back over the PAST WEEK, how often did you drink regular soda pop (not diet)? Seven response options ranged from "None" to "5 or more servings"	
Familial encouragement for healthful eating	During a typical week, how often have you or another member of your household encouraged your daughter to eat healthy foods? Five response options ranged from "Never" to "Every day" (19)	2-week test-retest *r* = .70
Frequency of family meals	During the past 7 days, how many times did all, or most, of your family living in your house eat a meal together? Nine response options ranged from "0 times" to "More than 7 times" (9)	2-week test-retest *r* = .74
Frequency of fast-food family meals	During the past 7 days, how many times was a family meal purchased at a fast-food restaurant (McDonald's, KFC, pizza, etc.) and eaten either at the restaurant or at home? Nine response options ranged from "0 times" to "More than 7 times" (20)	

a The question assessing the presence of a television in the adolescents' bedrooms was asked of the adolescent girls.
